# An efficient system for *Agrobacterium*-mediated transient transformation in *Pinus tabuliformis*

**DOI:** 10.1186/s13007-020-00594-5

**Published:** 2020-04-10

**Authors:** Shuangwei Liu, Jingjing Ma, Hongmei Liu, Yingtian Guo, Wei Li, Shihui Niu

**Affiliations:** Beijing Advanced Innovation Center for Tree Breeding By Molecular Design, National Engineering Laboratory for Tree Breeding, College of Biological Sciences and Technology, Beijing Forestry University, Beijing, 100083 People’s Republic of China

**Keywords:** *Pinus tabuliformis* callus, Efficient transformation system, *Agrobacterium*-mediated transformation, Transient gene expression, GUS staining

## Abstract

**Background:**

Functional genomic studies using genetics approaches of conifers are hampered by the complex and enormous genome, long vegetative growth period, and exertion in genetic transformation. Thus, the research carried out on gene function in *Pinus tabuliformis* is typically performed by heterologous expression based on the model plant Arabidopsis. However, due to the evolutionary and vast diversification from non-flowering (gymnosperms) to flowering (angiosperms) plants, several key differences may alter the underlying genetic concerns and the analysis of variants. Therefore, it is essential to develop an efficient genetic transformation and gene function identification protocol for *P*. *tabuliformis*.

**Results:**

In the present study we established a highly efficient transgene *Agrobacterium*-mediated transient expression system for *P*. *tabuliformis*. Using a β-glucuronidase gene (GUS) as a reporter gene expression, the highest transformation efficiency (70.1%) was obtained by co-cultivation with *Agrobacterium* strain GV3101 at an optical density at 600 nm of 0.8, with 150 μM acetosyringone for 30 min followed by 3 days in the dark at 23 ± 1 °C. This protocol would be applied to other conifers; GUS staining was observed 24 h post-infection.

**Conclusions:**

We report a simple, fast, and resilient system for transient *Agrobacterium*-mediated transformation high-level expression of target genes in *P*. *tabuliformis*, which will also improve transformation efficiency in other conifer species.

## Background

*Pinus tabuliformis* belongs to conifer species, native to northern and central China that has considerable economic and ecological valuable forest plants [1–4]. Functional genomic research in *P*. *tabuliformis* is typically performed by heterologous expression based on the Arabidopsis as a model plant. However, due to the evolutionary and genetic developmental divergence leap from non-flowering (gymnosperms) to flowering (angiosperms) plants, and key differences may alter the underlying gene expression and genetic programs [5]. Therefore, it is essential to develop an efficient genetic transformation and gene-function identification system for *P*. *tabuliformis*. The *Agrobacterium*-mediated method, including stable transformation and transient gene expression, has been functional in number of plant species [6–10]. Although stable and resilient transformation protocols has been successfully carried out in a few conifer species, such as *P*. *massoniana* [11], Korean fir [12], Slash pine [13], Maritime pine [14], Norway spruce, and Loblolly pine [15]. However, there is still major technical obstacle for most conifers. Transient genetic transformation is a simple and rapid technique for investigating protein localisation and gene function [16], and enables high-throughput analysis [17, 18]. Among various methods, Transient expression methods including particle bombardment, protoplast transformation using polyethylene glycol, electroporation, and *Agrobacterium*-mediated transformation have been used in conifer plants [19]. Transgenic plants have been produced by particle bombardment in *Larix gmelinii* [20], Norway spruce [21], Radiata pine [22, 23] and Black spruce [24]. Transient expression was observed in electroporated protoplasts of Douglas fir and Loblolly pine [25, 26]. Compared to commonly used particle bombardment and protoplast transformation, *Agrobacterium*-mediated transformation is used more frequently in many plant species. This method is one such versatile simple, rapid and transient expression of target genes can be detected within a few hours [8, 16, 27]. However, the average transformation efficiency is affected by the *Agrobacterium* strain [13], explant type [28], *Agrobacterium* density [29], acetosyringone (AS) concentration [30], and time period [11]. In this study, we obtained a hypocotyl-derived callus from *P*. *tabuliformis* seedlings, which promoted transient transformation. We developed a highly efficient and buoyant *Agrobacterium*-mediated transient transformation system for *P*. *tabuliformis* and assessed the influence of *Agrobacterium* density, infection time, AS concentration, co-cultivation duration, and sonication. The transformation system for *P*. *tabuliformis* in combination with high-throughput sequencing technologies are capable to improve the transformation efficiency for other conifer species.

## Methods

### Plant materials and treatments

Dry and mature seeds close to natural dispersal of *P*. *tabuliformis* were collected from first-generation clonal seed orchard located in Pingquan City, Hebei Province, China (GPS recordings; 40° 99′ N, 118° 45′ E, 560 m above sea level) and stored in plastic bags at 4 °C with optimal storage conditions. Seeds were spread out on sphagnum moss, soaked with water and germinated in a growth chamber under a 16/8 h (light/dark) photoperiod at 23 ± 2 °C. After 20 days. Seedlings were sterilised with 75% ethanol (v/v) for 1 min and rinsed three times with sterile distilled water. Further seedlings were cleaned in 17% NaClO (v/v) for 10 min followed by rinsing three to five times in double distilled water. Subsequently, the roots were removed by using a sterilised scalpel under aseptic conditions. The callus induction medium supplemented with hormones is prepared. The pH of the medium was adjusted to 5.8 prior to the addition of 8 g/L agar and autoclaving at 121 °C for 20 min. Finally, the hypocotyls with needles were inoculated on callus induction medium, [31] (Table 1) under 14/10 h (light/dark) photoperiod at 23 ± 2 °C. After approximately 40 days, yellow or green granular calli were transferred to fresh proliferation medium (Table 1). The calli were subcultured every 2–3 weeks on fresh callus proliferation medium. After four subcultures in the dark, yellow and soft calli were harvested as explants for transformation.

**Table 1:** Composition of different media used in the study

Media	Composition
Induction medium	1/2 MS, 1 mg/L dichlorophenoxyacetic acid (2,4-d), 3 mg/L 6-benzylaminopurine (6-BA), 30 g/L source (W/V), 8 g/L agar
Proliferation medium	DCR medium, 3% sucrose, 1.13 mg/L 6-BA, 2.21 mg/L 2,4-d and 3 g/L phytagel
Co-cultivation medium	DCR medium, 2.5% sucrose, 1.13 mg/L 6-BA, 2.21 mg/L 2,4-d and 3 g/L phytagel
Suspension medium	10 mM MES, 10 mM Mgcl_2_, 0.005% Tween20 and 50, 100, 150, 200 µM AS

### Plasmid construction and *Agrobacterium* culture

**Fig. 1:**
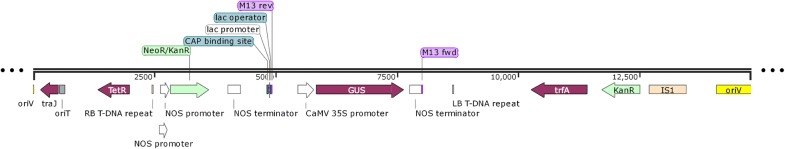
Map of the linear pBI121 plasmid. *GUS* driven by the CaMV 35S promoter, *nptII* under the control of the Nos promoter as a selectable marker, and the left (LB) and right (RB) border of T-DNA are shown

The plant binary vector pBI121 plasmid (Fig. 1) was induced into *A*. *tumefaciens* LBA4404 and GV3101 [32] via the freeze/thaw method. We selected a single colony of *Agrobacterium* cultured overnight in liquid YEB medium containing 50 mg/L kanamycin and 50 mg/L rifampicin (Sigma-Aldrich), followed by incubation with shaking at 180–220 rpm at 28 °C. Then the *Agrobacterium* cells were harvested by centrifugation at 5000 rpm for 8 min and the cells were rinsed twice in suspension solution containing 10 mM MES (pH 5.6), 10 mM MgCl_2_, and 0.005% Tween-20. Two transformation approaches were used; I, cells were suspended in the above liquid medium with same 150 µM AS to optical density at 600 nm (OD_600_) of 0.2, 0.4, 0.6, 0.8, and 1.0, respectively; II, cells were suspended to OD_600_ of 0.8 and add 50, 100, 150, and 200 µM AS, respectively. Finally, the suspension was placed at 23 °C for 3 h for transformation.

### *Agrobacterium*-mediated transient transformation

The pre-cultured calli were immersed in the *Agrobacterium* suspension and treated as in the following steps: (1) Calli were co-cultured with suspension solution (OD_600_ = 0.2, 0.4, 0.6, 0.8, and 1.0) for 30 min containing of 150 µM AS. (2) Calli were then infected by suspension solution for 10, 20, 30, and 40 min at OD_600_ of 0.8 in the presence of 150 µM AS. (3) Calli were treated by suspension solution (50, 100, 150, and 200 µM AS) for 30 min at an OD_600_ of 0.8. (4) at the end calli were placed in 50 mL sterile tubes containing 20 mL of *Agrobacterium* suspension for 30 min at OD_600_ of 0.8 in the presence of 150 µM AS. Subsequently calli were rinsed three times with sterile distilled water, resuspended in sterile water, and placed in a float at the centre of a sonicator bath. The sonicator was controlled by an electronic timer at a power of 100 W. The calli were sonicated for 5, 10, and 15 min and shaken twice at 5 min intervals. (5) Calli suspended in sterile water in 50 mL sterile tubes were sonicated for 5, 10, and 15 min and infected by suspension solution for 30 min at an OD_600_ of 0.8. After treatment, the infected calli were blotted with sterile filter paper to remove excess bacteria and cultured on co-cultivation medium with sterile filter paper in the dark at 23 ± 1 °C for 1–5 days. The hypocotyl seedling or needles were cut cross-sectionally into 2 cm long longitudinal fragments for absorption by *Agrobacterium* and were infected as in step 3, followed by, hypocotyls and needles were placed on two layers of wet cheesecloth in Petri dishes in the dark at 23 ± 1 °C for 3 days [8]. The calli were subjected to β-glucuronidase (GUS) staining daily.

### GUS staining assays

The transgenic calli, needles, and hypocotyls were submerged in GUS staining solution containing 50 mM sodium phosphate (pH 7.0), 0.5 mg/L 5-bromo-4-chloro-3-indolyl-β-dglucuronide, 0.1% Triton X-100, 0.5 mM K_3_[Fe(CN)_6_], and 0.5 mM K_4_[Fe(CN)_6_] overnight in the dark at 37 °C. The samples were washed several times in 75% ethanol to remove chlorophyll [33]. A blue colour in tissue was regarded as indicative of positive transgenic explants.

### Statistical analysis

Statistical analysis of the callus growth rate and GUS strain frequency of calli, needles, and hypocotyls was performed by Student’s t-test (p < 0.01). Infected calli were transferred to three Petri dishes (replication) per treatment, at nine calli per plate. The transformation efficiency (%) (number of positive calli, hypocotyls, and needles/ total number of infected calli, hypocotyls, and needles × 100%) was calculated. Each experiment was repeated three times independently.

## Results

### Callus induction and proliferation in *P*. *tabuliformis*

**Fig. 2:**
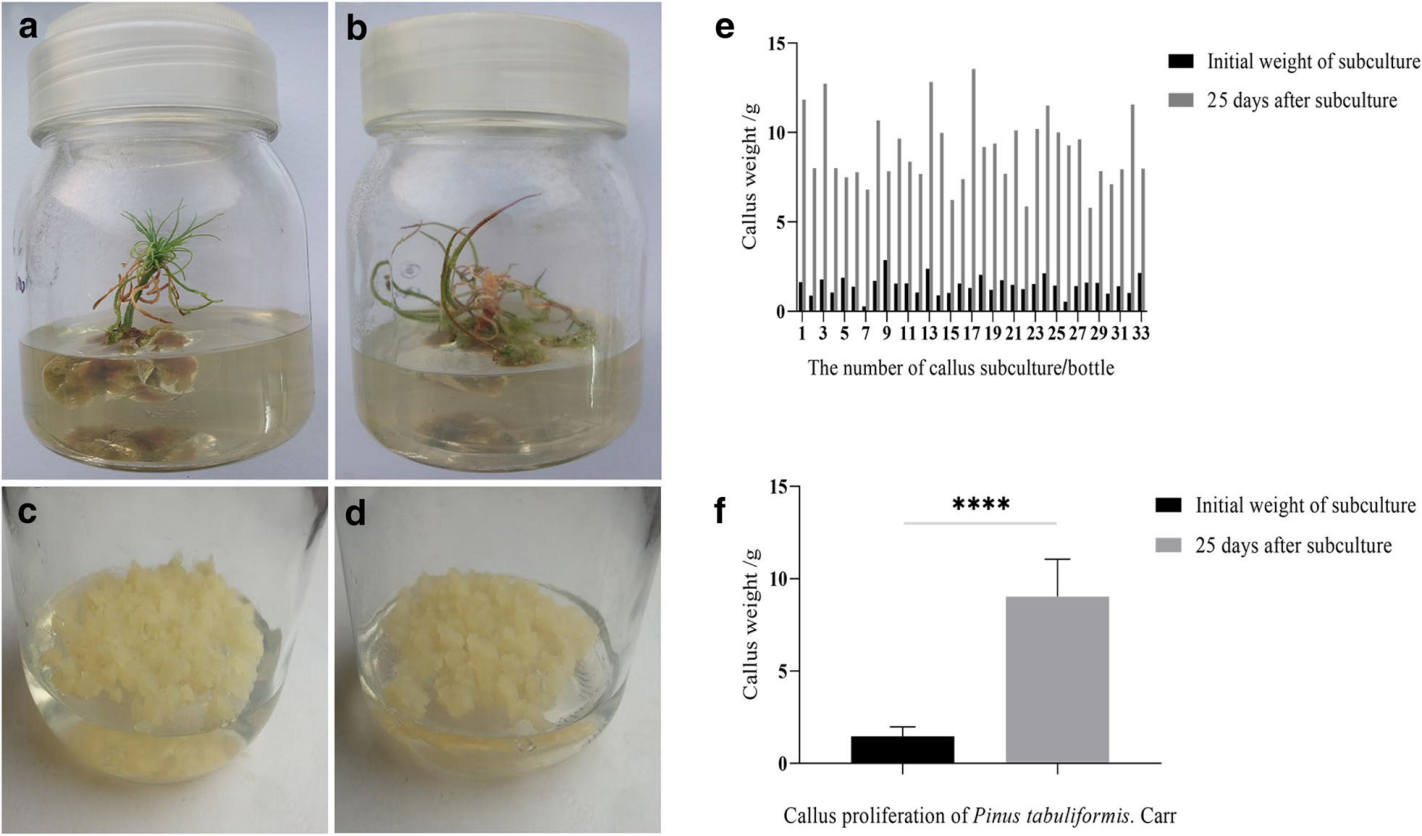
Callus induction and proliferation in *P*. *tabuliformis*. **a**, **b**, Roots were placed on induction medium. **c**, **d**, Calli were inoculated on proliferation medium. **e**, **f**, Growth rate of calli after 25 days. *Significant by Student’s *t*-test (p < 0.05)

The mature seeds were germinated in a growth chamber under ambient environmental conditions; 16/8 h (light/dark) photoperiod at 23 ± 2 °C. After 20 days of seed germination, hypocotyls of *P*. *tabuliformis* seedlings were cultured on callus induction medium (Table 1). Green callus tissues were developed on hypocotyl segments, followed by 4–5 weeks under light/dark (16/8 h) conditions (Fig. 2a, b). The calli were transferred into proliferation medium (Table 1) and subcultured on fresh medium subsequently every 3 weeks and kept under dark condition. A rapidly proliferating callus was obtained after several subcultures and hypocotyl-derived calli with a texture of pale yellow, smooth surface, a loose structure, and rapid growth were generated after 18–25 days (Fig. 2c, d). The average weight of calli was Three-twofold higher than the initial value after sub-culturing (Fig. 2e). The total weight increased significantly than callus induction weight (Fig. 2f). Sufficient materials for transient transformation were obtained at 18 days post-subculture.

### *Agrobacterium*-mediated transient transformation protocol for callus

**Fig. 3:**
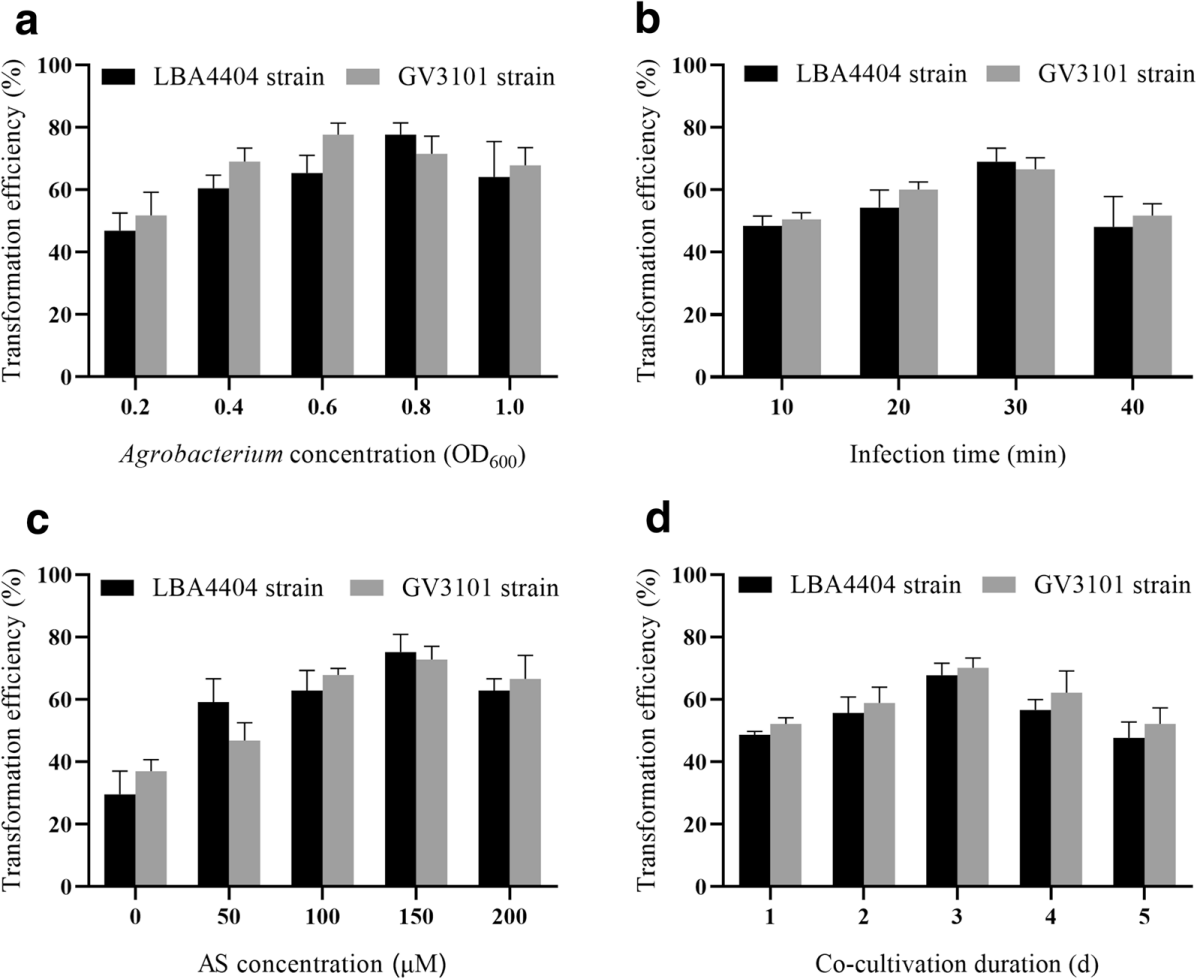
Effects of key factors on transient transformation using *A*. *tumefaciens* LBA4404 and GV3101. **a** Effect of *Agrobacterium* concentration on transient transformation (OD_600_ values of 0.2, 0.4, 0.6, 0.8, and 1.0). **b** Effect of infection time on transient transformation (10, 20, 30, and 40 min). **c** Effect of AS concentration on transient transformation (50, 100, 150, and 200 µM). **d** Effect of co-cultivation duration on transient transformation (1, 2, 3, 4, and 5 days). Each treatment comprised 27–30 explants and experiments were performed in triplicate. Data are mean ± SD

**Fig. 4:**
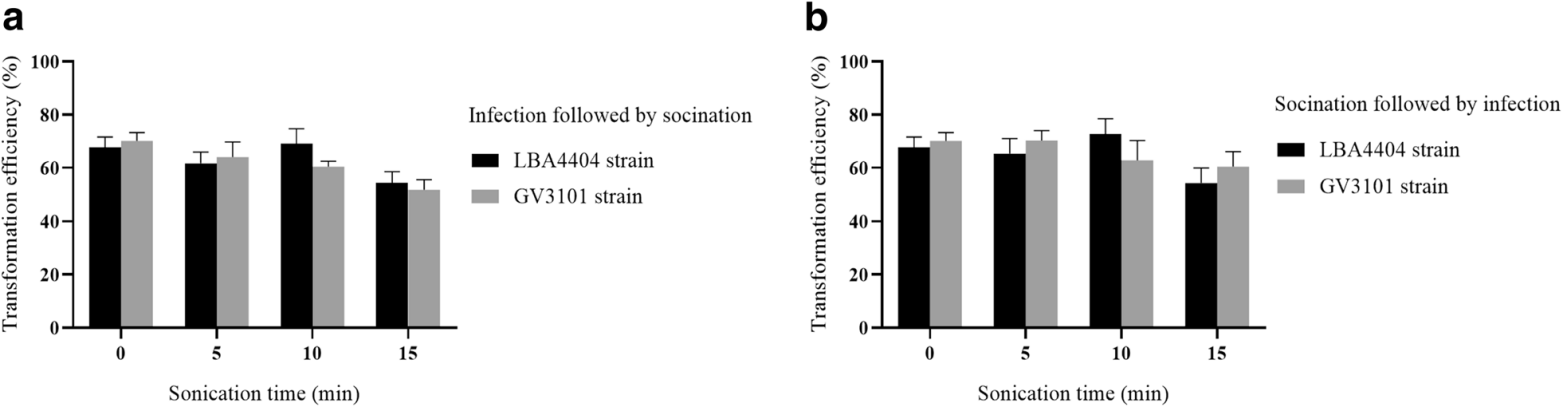
Effect of sonication on transient transformation using *A*. *tumefaciens* LBA4404 and GV3101. **a** Calli were infected for 30 min and sonicated (5, 10, and 15 min). **b** Calli were sonicated (5, 10, and 15 min) and infected for 30 min. Each treatment comprised 27–30 explants and was performed in triplicate. Data are mean ± SD

In the present study we evaluated the effect on transformation efficiency of *Agrobacterium* density, treatment time, AS concentration, co-cultivation duration, and sonication. The highest transformation efficiency was obtained with *Agrobacterium* strains GV3101 and LBA4404 at OD_600_ of 0.6 and 0.8, respectively (Fig. 3a). The largest number of positive GUS calli (77.7%) was obtained while calli were infected with *Agrobacterium* strain GV3101 at OD_600_ of 0.6 and with LBA4404 at OD_600_ of 0.8. The transformation efficiency was decreased at higher or lower concentration of OD_600_. The highest transformation efficiency recorded from infection with *Agrobacterium* GV3101 and LBA4404 for 30 min (Fig. 3b). A shorter infection time reduced transformation efficiency, and longer infection time might be damage calli. Therefore, we used an infection time of 30 min in subsequent experiments. AS induced the expression of virulence genes and enhances *Agrobacterium* infection of wound segments. The AS concentration significantly influenced by the transformation level efficiency; the highest efficiency of 75.2% (GV3101) and 72.7% (LBA4404) were obtained at 150 µM AS (Fig. 3c). Co-cultivation for 3 days post-infection resulted in the highest transformation efficiencies of 70.1% (GV3101) and 67.7% (LBA4404) (Fig. 3d). The transformation efficiency were decreased with increasing co-culture duration was triggered by overgrowth of *Agrobacterium*, leading to callus browning. We assayed the effect of sonication time by infecting calli with *Agrobacterium* for 30 min followed by sonication (IFS), and by sonicating calli followed by infection for 30 min (SFI) (Fig. 4). Sonication did not significantly influence the transformation efficiency using *A*. *tumefaciens* LBA4404 and GV3101.

### Application of the transient transformation protocol to other conifer species

**Fig. 5:**
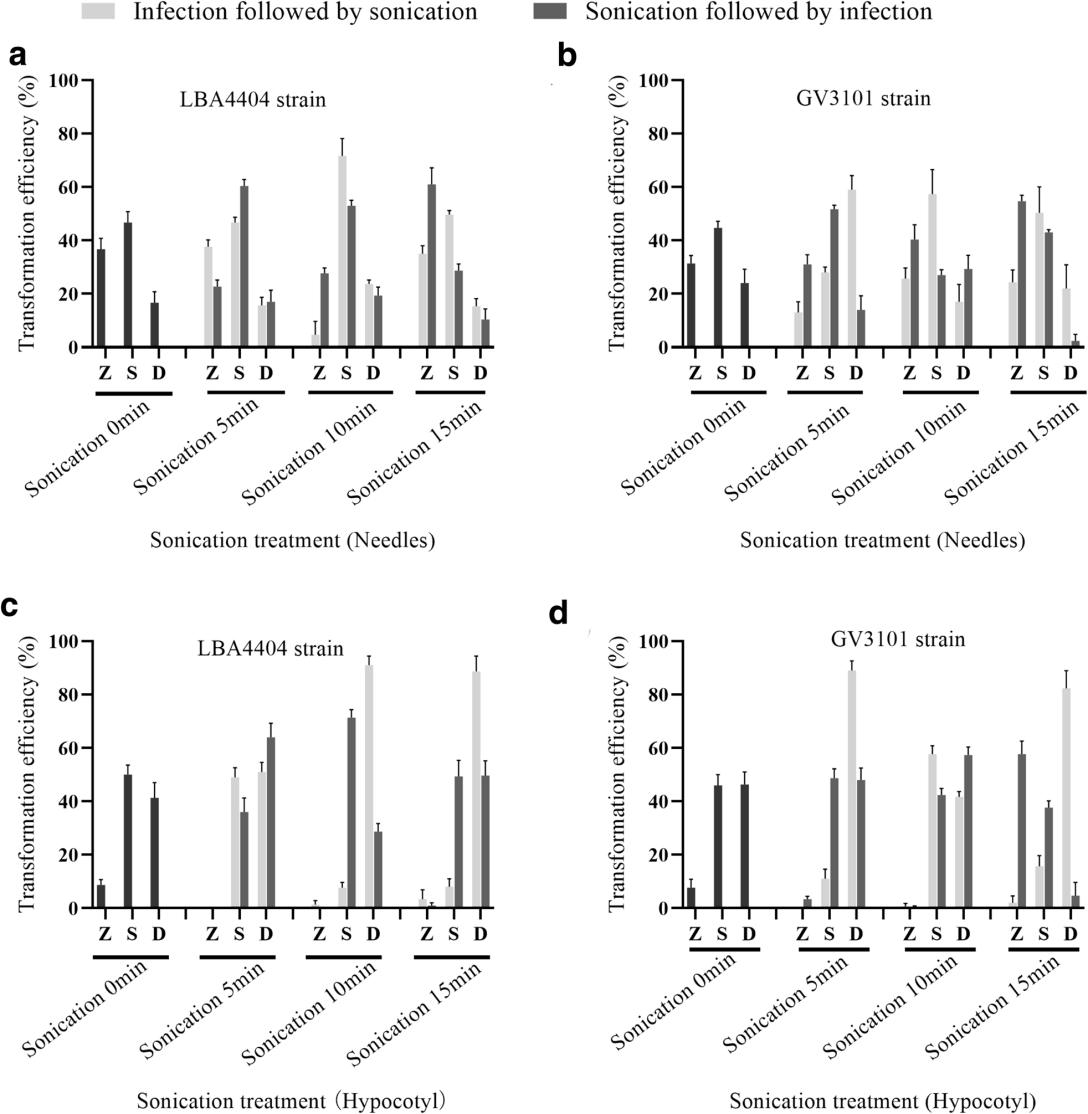
Influence of sonication on transient transformation efficiency in explants of *P*. *tabuliformis*. **a** Effect of sonication on transient transformation using *A*. *tumefaciens* LBA4404. Needles were infected for 30 min and sonicated (5, 10, and 15 min). **b** Effect of sonication on transient transformation using *A*. *tumefaciens* GV3101. Needles were sonicated (5, 10, and 15 min) and infected for 30 min. **c** Effect of sonication on transient transformation using *A*. *tumefaciens* LBA4404. Hypocotyls were infected for 30 min and sonicated (5, 10, and 15 min). **d** Effect of sonication on transient transformation using *A*. *tumefaciens* GV3101. Hypocotyls were sonicated (5, 10, and 15 min) and infected for 30 min. z, no GUS expression in needles or hypocotyl fragments; s, GUS expression in single-end needle or hypocotyl cross-sections; d, GUS expression in double-end needle or hypocotyl cross-sections. Each treatment comprised 100 explants and was performed in triplicate. Data are mean ± SD

**Fig. 6:**
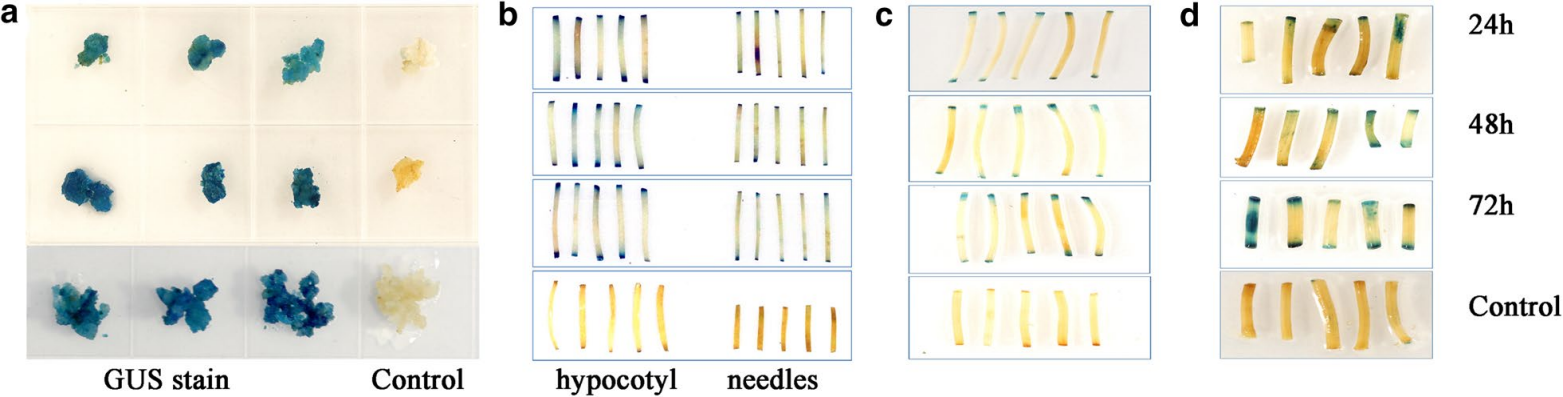
Histochemical assay of GUS expression in organs of *P*. *tabuliformis*, *P*. *yunnanensis*, and *Picea crassifolia* transformed by agroinfiltration. GUS expression was examined in organ cross-sections at 24, 48, and 72 h after infection. **a** Callus and **b** hypocotyl and needles of *P*. *tabuliformis*. **c** Hypocotyl of *Picea crassifolia*. **d** Hypocotyl of *P*. *yunnanensis*. Negative controls (without pBI121). Each treatment comprised 30 explants and was performed in triplicate

The transient transformation system in needles of *P*. *tabuliformis* and in seedlings and hypocotyls of *P*. *tabuliformis*, *P*. *yunnanensis*, and *Picea crassifolia* Kom were tested. (Fig. 6) The tissues were immersed in suspensions of *Agrobacterium* GV3101 and LBA4404 as described above. First, we assessed the transformation efficiency in needles and hypocotyls of *P*. *tabuliformis* seedlings (Fig. 5) using the IFS and SFI approaches. Sonication under both IFS and SFI conditions enhanced the staining efficiency. However, in case of needles, there was no significant difference between IFS and SFI. The transformation efficiency was observed greater while using single than double ends; the highest efficiency was 71.66% (single end) using LBA4404 by SFI. In hypocotyls, IFS exhibited a higher transformation efficiency than SFI when explants were infected and sonicated for 5–10 min (Fig. 5c, d). The highest transformation efficiency was recorded with *Agrobacterium* LBA4404 after sonication for 10 min for hypocotyls (91% [double ends]) (Fig. 5c).

These results indicated that *Agrobacterium* was more readily infected hypocotyls than needles and that was not the same case as in callus. GUS staining showed that the optimised protocol is also appropriate for the hypocotyls and needles of other conifer species. Particularly, GUS activity in the hypocotyls of *P*. *tabuliformis* seedlings was detected 24 h post-infection (Fig. 6b). Therefore, we tested the GUS activity of explants at 24, 48, and 72 h post-infection. The results suggested that the agroinfiltration system could be used for other conifer species (*P*. *tabuliformis*, *P*. *yunnanensis*, and *Picea crassifolia*) and most hypocotyls showed GUS activity at 24 h post-infection (Fig. 6c, d). Explants were infected and sonicated for 5–10 min for transformation of hypocotyls and needles of *P*. *tabuliformis* seedlings. The optimised transformation system was also suitable for other conifer species.

## Discussion

*Agrobacterium*-mediated transient transformation was used in many angiosperm plants to get transient and high-level expression of target genes, such as in Arabidopsis, rice, wheat, *Nicotiana benthamiana*, strawberry, and soybean [7, 34–37]. The first successful *Agrobacterium*-mediated transformation of conifer was in Sugar pine has the longest cones of any conifer. [38]. The method has since been extensively used in other conifer species, including Norway spruce, Loblolly pine [15], White pine [8], *P*. *massoniana* [11], Slash pine [13], *P*. *patula* [39], and *P. radiata* [40]. Use of embryos, cotyledons, hypocotyls, and female gametophytes as explants reportedly induces non-embryogenic or embryogenic calli and adventitious buds [41–44]. Mature or immature zygotic embryos were thought to be ideal transformation materials for generating embryonic tissues. However, the process was complex and was influenced by genotype, explant type, developmental stage, and medium composition [45]. Therefore, the production and regeneration of conifers by somatic embryogenesis is difficult and slow as compared to other plant species [45, 46], which hampering the development of transformation systems for conifer species. In this study, hypocotyls with needles from *P*. *tabuliformis* seedlings were used as explants to induce calli. Callus from *P. tabuliformis* seedlings didn’t affected by seasonal variation and can be frequently stable. The hypocotyl-derived calli enabled more rapid and efficient transformation than the embryogenic calli. The calli showed a high growth rate at 25 days post-subculture, and thus *Agrobacterium*-mediated transient transformation of *P*. *tabuliformis* could be completed in < 1 month. Furthermore, hypocotyl-derived calli maintained rapid growth for > 2 years, suggesting the method to be suitable for routine large-scale transformation using *Agrobacterium*. Moreover, there are many factors contribute to affect the transformation efficiency of plants. *Agrobacterium* LBA4404 and GV3101 have been used for transformation of Slash pine, *P*. *radiata*, *P*. *patula,* Loblolly pine, and Norway spruce [13, 15, 26, 28, 39, 40, 47]. In this study, GV3101 showed a higher transformation efficiency than LBA4404 (Fig. 5). Consistently, transient transformation of *P*. *tabuliformis* hypocotyl and needles using GV3101 resulted in stronger GUS activity (Fig. 6). An excessively high *Agrobacterium* concentration damages explants, leading to browning and death of calli. By contrast, an insufficient *Agrobacterium* concentration results in a low rate of infection [48]. The highest transformation efficiency using *Agrobacterium* GV3101 and LBA4404 was OD_600_ of 0.6 (77.7%, GV3101) and 0.8 (77.73%, LBA4404), respectively, as reported previously [13, 49]. Therefore, we used an *Agrobacterium* suspension with OD_600_ of 0.8 and an infection duration of 30 min (Fig. 2b). Regarding the AS concentration, the highest transformation efficiency was detected at 150 µM AS. Because we did not add antibiotics to the medium, *Agrobacterium* overgrowth with increasing co-cultivation duration caused browning and death of calli, which was consistent with the observed lower transformation efficiency. The efficiency of T-DNA delivery into the host cell affects *Agrobacterium*-mediated transient transformation, and sonication reportedly enhances transformation of calli of several plant species, such as Slash pine [13], this might be due to the cell membrane permeability. However, sonication for 5, 10, and 15 min did not significantly improve transformation efficiency. This result is not in agreement with a prior report on Slash pine [13], likely because prolonged sonication caused damaged to the calli. Furthermore, strong GUS expression was observed in needles and hypocotyls of *P*. *tabuliformis* and hypocotyls of *P*. *tabuliformis*, *P*. *yunnanensis*, and *Picea crassifolia*. The transformation efficiency was higher in hypocotyls than needles was recorded. Therefore, the efficiency of *Agrobacterium*-mediated transformation in pine was dependent on the explants used. We established and optimised an *Agrobacterium*-mediated transformation system, which will enable studies of functional genes in *P*. *tabuliformis* and in other conifer species.

## Conclusion

The *P*. *tabuliformis* genome has not been sequenced due to large size (~ 25.6 Gb) and generating transgenic *P*. *tabuliformis* plants is problematic. Thus, to identify functional genes in *P*. *tabuliformis*, a rapid and efficient *Agrobacterium*-mediated system was developed. We transformed hypocotyl-derived calli to enable transient expression in *P. tabuliformis*. The transformation process includes callus proliferation required only 2–3 weeks and also suitable/recommended for other conifer species. This protocol enables rapid establishment of transgenic calli and provides the materials needed to study gene functions in *P*. *tabuliformis* calli.

## Data Availability

The datasets used and/or analyzed during the current study are available from the corresponding author on reasonable request.
